# Italian Legal Euthanasia: Unconstitutionality of the Referendum and Analysis of the “Italian” Problem

**DOI:** 10.3389/fsoc.2022.898783

**Published:** 2022-07-12

**Authors:** Maricla Marrone, Pietro Berardi, Biagio Solarino, Davide Ferorelli, Serena Corradi, Maria Silvestre, Benedetta Pia De Luca, Alessandra Stellacci, Alessandro Dell'Erba

**Affiliations:** ^1^Section of Legal Medicine, University of Bari, DIM, Bari, Italy; ^2^Department of Anesthesia and Resuscitation, Di Venere Hospital, Bari, Italy

**Keywords:** referendum, assisted suicide, euthanasia, administration of palliative medication, European context

## Abstract

The term “euthanasia” refers to medical interventions that involve the direct administration of a lethal drug to the patient who requests it and meets certain requirements. Currently in Italy euthanasia constitutes a crime and falls within the hypotheses foreseen and punished by Article 579 (Murder of consenting person) or by article 580 (Instigation or aid to suicide) of the penal code. On the contrary, medically assisted suicide in some cases and the suspension of treatment constitute an inviolable right pursuant to art. Thirty two of the Constitution and Law 219/2017. Thanks to the sentence 242/2019 of the Constitutional Court, in Italy it is instead possible to request medically assisted suicide, that is, the indirect help of a doctor to die. There are four conditions required: whoever requests it must be fully capable of understanding and willing, must have an irreversible pathology that is the bearer of severe physical or mental disease, and must survive thanks to life-saving treatments. The Italian referendum “Free until the end” aims to introduce legal euthanasia through the partial repeal of art. 579 c.p. which punishes the murder of the consenting party. The authors analyze the reasons for the referendum in the light of the Italian and European scenario, analyzing the first Italian case of assisted suicide immediately after the referendum which inevitably becomes a starting point for ethical and medico-legal reflection on the issue. On 02.15.2022 the Italian Constitutional Court declared the Referendum on Legal Euthanasia inadmissible.

## Introduction

The term “euthanasia” derives from ancient Greek and literally means “good death”; it is a polysemic term, that is, with different meanings, even if etymologically related (Möller, 2021).

In general, it represents the act by which a doctor or other individual administers drugs, at the free request of the informed and informed subject, to intentionally cause the immediate death of the applicant. The goal of the act is to anticipate death and shorten the agony of a terminally ill patient upon request; in this sense, it can be placed in the more general case of the murder of the consenting party (art. 579 of the Italian Criminal Code).

In other words, it represents the clinical practice with which to cause the death of an individual whose quality of life is permanently compromised by an illness, impairment, or mental condition.

We are talking about:

- Administration of palliative medication (passive euthanasia), when the doctor refrains from taking care of keeping the patient alive;
- Euthanasia when the doctor directly causes the patient's death.

Discontinuation of treatment in very severe cases and/or administration of palliative medication is allowed almost everywhere, for example in the Netherlands, Belgium, France, Sweden, Germany (Julesz, 2013; Cuman and Gastmans, 2017).

Much more circumscribed is the perimeter of the European states that have regulated assisted suicide, in which medical and administrative help brought to a person who has decided to die by suicide is limited to the assistance of the person for the aspects of hospitalization, preparation of substances, post mortem technical and legal management, fully entrusting the deed to the subject. Despite some differences, today it is only regulated in the Netherlands, Belgium, Luxembourg, Switzerland, and Spain.

Another case is the help or assistance to suicide, which differs from euthanasia because in this case it is the person who carries out the last act that causes his death, an act made possible thanks to the decisive collaboration of a third party, who can also be a doctor, who prescribes and delivers the lethal product for a certain period and under the strict conditions set by the legislator.

There are cases in which the procedure uses machinery that can help the patient with reduced physical capacity to take the lethal product prepared (by the doctor or by others). For the most part, suicide aid is provided with the assistance of a doctor, pharmacist or nurse and within the care facilities (medical suicide aid).

In Italy, no form of euthanasia is allowed, much less the use of assisted suicide; in our country euthanasia is similar to voluntary murder even if the patient is consenting and, therefore, it is a crime.

In this regard, article 580 of the Criminal Code does not distinguish the hypothesis of instigation and aid to suicide and states: “Anyone who causes others to commit suicide or reinforces the purpose of suicide, or facilitates its execution in any way, is punished, if it occurs suicide, with imprisonment from 5 to 12 years. If the suicide does not take place, he is punishable by imprisonment from 1 to 5 years, provided that the attempted suicide results in a serious or very serious personal injury.”

In this way, the legislator has decided to safeguard the unavailability of a very personal and immanent good such as life and, therefore, to punish the conduct of the subject that determines or strengthens the purpose of others to put an end to their existence.

However, the current regulatory framework for the end of life seems to leave some situations equally deserving of protection and respect without adequate protection, those in which the value of the protection of human life must necessarily be in balance with other constitutionally relevant goods. These are certainly not easily predictable events in the last century and which were created following the development of the skills of medicine, which on the one hand can save the lives of patients in serious clinical conditions, but on the other, some of them are then “Forced” to live in a state of irreversible debilitation, sometimes even of dependence and technological suffering, which can lead patients to consider their life undignified.

## European Perspective

After the legalization of euthanasia and assisted suicide (EAS) in the Netherlands in 2002, movements began to implement similar laws in other European countries (Onwuteaka-Philipsen et al., 2012; Chao et al., 2016).

The multiplicity of European legislation on euthanasia fits into a very heterogeneous framework, with severe countries, on the one hand, which punish all forms of assisted suicide such as Ireland, Greece and Romania, and on the other countries such as Belgium and the Netherlands has also authorized euthanasia for children under 18 (the latter, however, only for infants and those over 12).

THE NETHERLANDS were the first country in Europe to amend its penal code in 2001 to make direct euthanasia and assisted suicide legal under strictly regulated circumstances.

The “Law for the control of interruption of life on request and assistance in suicide,” definitively approved by the Dutch Parliament on 10 April 2001, allows the euthanasia doctor a terminally or chronic ill who expressly requests it and to assist a patient who decides to take his own life. There are two prerequisites for such a medical intervention: any possible therapy must have been discussed and there must be a pressing and unequivocal (spontaneous and thoughtful) request from the patient to put an end to his life, expressed in the ways and forms required by law about the state and age of the patient.

To ensure the correct use of this standard, a regional Commission composed of qualified experts (a jurist with the functions of president, a doctor, and an expert in bioethics) monitors the behavior of doctors in specific cases that must be reported to them by health professionals.

If the doctor ends the life sentence on request, the law in question provides that the death certificate must be drawn up with the indication of “unnatural” death.

In 2005, the “Groningen Protocol” was approved. It regulates life termination for infants in their first year. Present governmental initiatives have been taken to extend this to all minors under 12 years, subject to parental authorization.

Soon after, more precisely in 2002, it was BELGIUM that legalized euthanasia and extended the scope to minors in 2016 (Cuman and Gastmans, 2017). The patient's rights law (Loi Belge relative aux droits du Patient) explicitly admits the right to refuse hydration and artificial nutrition.

Every capable adult or emancipated minor can, if he is no longer able to express his will, in writing in a declaration, indicate his intention to be killed, provided he is suffering from a serious, incurable and irreversible accidental or pathological disease.

As regards the form, the Belgian legislation specifies that the advance declaration can be made at any time; however, its validity requires that it be drawn up in the presence of two adult witnesses, of which at least one has no material interest in the death of the declarant (Saad, 2017).

In LUXEMBOURG, parliament passed the law legalizing euthanasia in 2008, making it the third country in the European Union to decriminalize euthanasia. The ill can choose to end their life after receiving prior approval from a medical team of experts (Julesz, 2014).

Swiss law allows for both indirect euthanasia (taking substances whose side effects can shorten life span), administration of palliative medication (disruption of care and maintenance devices in life) and assisted suicide. The latter is practiced outside the state health institutions by some associations, which accept applications from the terminally ill and suffering, regardless of the nationality of the applicant.

Recently, SPAIN joined this list, where a law in favor of euthanasia was approved by Parliament on March 18, 2021. The law governs both euthanasia—described as “the administration of a substance to the patient by a competent health worker”—and medically assisted suicide, “the prescription or administration by a health professional of a substance to the patient, so that the latter can take it autonomously, to cause its death” (Bernal-Carcelén, 2020).

In FRANCE there is the “Loi relative aux droits des maladies et è la fin de vie,” better known as “loi Leonetti,” which provides that everyone has the right to be informed about their state of health and that the doctor must respect the wishes of the person, after having informed them of the consequences of their choice. In any case, the patient is required to confirm his will after a suitable time interval, the so-called cooling period (“cooling period”), typical of withdrawal procedures (Toporski et al., 2017).

In the UK, suicide aid is prosecuted under the Suicide Act of 1961, although there is room for administration of palliative medication in case law and jurisdiction.

As for the other European countries:

• SWEDEN (since 2010), FINLAND (since 2012) and NORWAY authorize administration of palliative medication, provided that the interested party submits an adequate application, same scenario, for HUNGARY and IRELAND.

• In GERMANY administration of palliative medication and assisted suicide of a terminally ill patient or already in a coma do not constitute a crime, provided that the patient understands and desires it and explicitly requests it. The German “Bundestag,” approved on June 18, 2009, governs the provisions of patients based on the right to therapeutic self-determination, including the right to refuse medical treatment. Supreme Court first allowing individuals to assist in suicide in incidental cases, in 2021 allowing organized assisted suicide (Schreiber, 2008).

• In PORTUGAL, on the other hand, the Ethics Council has admitted, only in some cases, the exclusion of hydration and nutrition.

• In AUSTRIA and DENMARK, euthanasia is not allowed in any form. However, Austria is on the bring of allowing assisted suicide.

## Italian Scenario: the Roots of the Referendum

Thanks to the civil disobedience of Marco Cappato and Mina Welby^1^, in 2017 Italy obtained the law that recognizes the value of the Biological Testament, and a sentence of the Constitutional Court that opened gaps in the availability of human life.

Today in Italy only patients for whom the interruption of therapies is sufficient, as required by Law 219/2017, can put an end to their suffering (“Gazzetta Ufficiale, 2021).

The Constitutional Court has clarified that assistance in suicide (art. 580 of the Criminal Code) is not punishable if those who request it are kept alive with life-saving treatments.

All other people with irreversible pathologies that cause intolerable pain, and patients unable to take a drug on their own (for ALS, quadriplegia…) in our country cannot choose, and ask for medical assistance for voluntary death, because ours Criminal Code prohibits the murder of the consenting party (Article 579 of the Criminal Code).

### The Italian Referendum for Legal Euthanasia

The murder of the consenting party, governed by art. 579 of the Criminal Code, is a special offense (with respect to that provided for by art. 575 of the Criminal Code on murder) inserted, by the legislator, among crimes against life and individual safety. In order for the crime to take place, the victim's consent must be personal, effective, serious, explicit and unequivocal. Furthermore, it must be valid and without reservations, it can be given in any form as long as it is unconditional and existing up to the moment in which the fact is committed, this consent being revocable at any time. Although the good of life is considered in the Italian legal system as unavailable, the legislator wanted to recognize the lesser incisiveness of the murder of the consenting party. The rule in scrutiny protects the good of life even against the will of the owner.

On 10.08.2021 1,239,423 signatures were deposited in the Supreme Court to ask for the referendum “free until the end”/“legal euthanasia.”

Of these signatures, 391,874 were collected online, the others-−847,549—instead in over a thousand municipalities. This is a result that goes well beyond the five hundred thousand signatures needed to request a popular referendum.

The Legal Euthanasia Referendum asked for the partial repeal of art. 579 of the penal code. Here is the text and related changes:

Anyone who causes the death of a man, with his consent, is punished with imprisonment from 6 to 15 years. The aggravating circumstances indicated in Article 61 do not apply. The provisions relating to murder [art. 575–577] apply if the offense is committed:

- 1. Against a person under the age of eighteen;
- 2. Against a mentally ill person, or who is in conditions of mental deficiency, due to another infirmity or the abuse of alcohol or drugs.
- 3. Against a person whose consent has been extorted by the offender with violence, threat or suggestion, or stolen with deception [art. 613 bis].

The referendum aimed not to discriminate against certain individuals who request the end of life. In fact, the current formulation requires, in order to carry out assisted suicide (different from euthanasia), that the subject is suffering from an irreversible disease and that he is kept alive by life-sustaining treatments, in addition to having explicitly refused palliative care.

So it excludes some types of patients, such as terminal cancer patient, not subjected to life-sustaining treatment, even though they are aware of having to wait for death, perhaps in conditions of unquantifiable suffering.

The judgment 242/2019 of the Consulta on the Cappato/Dj Fabo case, while opening under certain conditions to a lawful proceeding in the context of assisted suicide, allows the person to procure assisted death only independently, but if the latter does not want or cannot—due to a totally disabling disease—will be excluded from this right.

The Legal Euthanasia Referendum was declared inadmissible on 02.15.2022: the Constitutional Court declared that the reform on the end of life would not guarantee the necessary protection “of human life.”

## Ethical and Medical-Legal Considerations

Death is a borderline situation for the individual, for modern medicine, for society and culture.

Euthanasia, between the shortening of life and assistance in death, deeply touches the image that man has of himself and of the world, the concept of disease and death, of freedom and dependence, of nature, society, and culture. Euthanasia, as a result of technical and scientific progress, is a challenge for medicine and society, for the sick, but above all for doctors.

In the Hippocratic Oath, euthanasia and assisted suicide have a fundamental meaning: “Never, moved by the thoughtful insistence of anyone, will I offer lethal medicines or commit such things.” It follows that the doctor can only preserve and protect life without ever putting it in danger or putting it to an end. The Geneva Declaration of 1948 is also deeply linked to this affirmation: “I will maintain the utmost respect for human life from the moment of its conception.”

The chances of medicine for prolonging life and thus postponing death have increased astonishingly. Medical progress, however, cannot exclude one of the few certainties of human life: having an end and being aware of this end. Medicine must recognize this paradox, wanting to cure what is ultimately not curable. In his relationship with the dying, the doctor learns his limitations.

Opinions differ on the autonomous euthanasia desired by the patient with the medical intervention. More and more people are engaged in this possibility but doubts against legalization are still heavy. The repercussions on the doctor-patient relationship are important: the sick and dying can lose faith in medicine and in the maintenance of life on the part of doctors; in the case of euthanasia, we are already thinking of people who are not really in the final stage of their life.

The autonomy of the patient is opposed to that of some doctors. He must live with the plurality of ethical orientations and religious convictions; a minimum ethical consensus and its legal protection with the laws must be sought; where it is not possible to reach it, the patient must know the ethical orientation of the doctor who treats him or the competent hospital structure.

Death as a social phenomenon is strongly influenced by external aspects such as religious beliefs or the absence of beliefs, traditions, customs and habits with profound differences depending on the country.

Death is also an event for those who remain, for those who remember the person and his departure. Euthanasia is not only a question of individual autonomy but is closely linked to the reaction of the family and society to illness, disability and age.

While it is true that States have a legitimate interest in protecting people from irrational, misinformed, pressurized, or unstable decisions to hasten their death, on the other hand, it should regulate the death assistance that doctors can provide to terminal patients who are in excruciating pain, otherwise doomed to an existence they consider intolerable.

Balancing needs and expectations in such a delicate and ethically important contest is not easy, nor do the authors want to direct the opinion of readers in one direction or another; however, it is indubitable that, in the face of the greater awareness of individual patients and the cultural change in social and professional relationships, it will be inevitable to address and regulate the issue.

The fundamental future challenge for medicine, society and the State, as well as for the individual, must be sought in euthanasia as a psycho-spiritual accompaniment of the dying with possible analgesia.

A greater quantitative and qualitative significance must be attributed to the reflections on how to carry out accompaniment to death in medicine and outside it than the debate on the pros and cons of the legalization of euthanasia.

## Author Contributions

Conceptualization: MM. Methodology: AS. Software: MS. Validation: SC. Formal analysis: AD. Investigation: PB. Resources: BS. Data curation: DF. Writing-original draft preparation: BD, MS, and SC. Writing-review and editing: BD. Visualization: AD. Supervision: M.MEDICO. All authors have read and agreed to the published version of the manuscript.

## Conflict of Interest

The authors declare that the research was conducted in the absence of any commercial or financial relationships that could be construed as a potential conflict of interest.

## Publisher's Note

All claims expressed in this article are solely those of the authors and do not necessarily represent those of their affiliated organizations, or those of the publisher, the editors and the reviewers. Any product that may be evaluated in this article, or claim that may be made by its manufacturer, is not guaranteed or endorsed by the publisher.

## Footnotes

^1^Marco Cappato and Mina Welby were ≪accused of the crime p. and.p. by art. 580 c.p. for having strengthened the suicidal intention of Antoniani Fabiano (known as Fabo), suffering from quadriplegia and blindness following a road accident on 13 June 2014, offering him the possibility of obtaining assistance in suicide at the headquarters of the Dignitas association, in Plaffikon in Switzerland, and taking steps to put Antoniani's family in contact with Dignitas by providing them with information material; in addition, for having facilitated the suicide of Antoniani, transporting him by car to Dignitas on 25 February 2017, where the suicide occurred on 27 February 2017”.

## References

[B1] Bernal-Carcelén I. (2020). Euthanasia: trends and opinions in Spain. Rev. Esp. Sanid. Penit. 22, 112–115. 10.18176/resp.00020 33300934 PMC7754540

[B2] Chao Y. S. Boivin A. Marcoux I. Garnon G. Mays N. Lehoux P.. (2016). International changes in end-of-life practices over time: a systematic review. BMC Health Serv. Res. 16:539. 10.1186/s12913-016-1749-z 27716238 PMC5048435

[B3] Cuman G. Gastmans C. (2017). Minors and euthanasia: a systematic review of argument-based ethics literature. Eur. J. Pediatr. 176, 837–847. 10.1007/s00431-017-2934-8 28573404

[B4] Gazzetta Ufficiale (2021). Available online at: https://www.gazzettaufficiale.it/eli/id/2020/01/17/20G00005/sg (accessed November 22, 2021).

[B5] Julesz M. (2013). [Euthanasia]. Orv. Hetil. 154, 671–674. 10.1556/OH.2013.29576 23608315

[B6] Julesz M. (2014). [Passive euthanasia and living will]. Orv. Hetil. 155, 1057–1062. 10.1556/OH.2014.29950 24974840

[B7] Möller H.-J. (2021). The ongoing discussion on termination of life on request. A review from a German/European perspective. Int. J. Psychiatry Clin. Pract. 25, 2–18. 10.1080/13651501.2020.1797097 32729770

[B8] Onwuteaka-Philipsen B. D. Brinkman-Stoppelenburg A. Penning C. de Jong-Krul G. J. F. van Delden J. J. M. van der Heide A. (2012). Trends in end-of-life practices before and after the enactment of the euthanasia law in the Netherlands from 1990 to 2010: a repeated cross-sectional survey. Lancet 380, 908–915. 10.1016/S0140-6736(12)61034-4 22789501

[B9] Saad T. C. (2017). Euthanasia in Belgium: legal, historical and political review. Issues Law Med. 32, 183–204. 29108142

[B10] Schreiber H.-L. (2008). [Legislative initiatives concerning euthanasia]. Z. Evid. Fortbild. Qual. Gesundhwes. 102, 208–214. 10.1016/j.zefq.2008.02.050 19004185

[B11] Toporski J. Jonveaux-Rivasseau T. Lamouille-Chevalier C. (2017). [End-of-life debate: Citizen's point of view about deep and continuous sedation]. La Rev. Med. Interne 38, 800–805. 10.1016/j.revmed.2017.09.008 29102388

